# A Systematic Review and Meta-Analysis: Does Braun Anastomosis Improve Billroth II Reconstruction After Distal Gastrectomy?

**DOI:** 10.7759/cureus.100856

**Published:** 2026-01-05

**Authors:** Mustafa Smisim, Mohammed Alwahibi, Naif F Aljamlan, Rakan S Shaheen, Mohamad Hamdan, Diyaa M Khalaf, Abdulrazzak A Al jaja, Sara W Alhabeeb, Manar A Alroidan, Mohammed H Sbaih

**Affiliations:** 1 College of Medicine, King Saud University, Riyadh, SAU; 2 Department of Surgery, Saudi German Hospital, Riyadh, SAU; 3 Department of Medicine, Dar Al Uloom University, Riyadh, SAU; 4 General Practice, First Specialized Medical Center, Riyadh, SAU; 5 Faculty of Medicine, International University of Africa, Khartoum, SDN; 6 General Practice, King Fahd Hospital, Al-Ahsa, SAU; 7 Department of Surgery, Prince Mohammed Bin Abdulaziz Hospital, Riyadh, SAU

**Keywords:** bile reflux, billroth ii reconstruction, braun anastomosis, distal gastrectomy, gastric cancer

## Abstract

For various surgical indications, distal gastrectomy with Billroth II (B-II) reconstruction is a common technique. Still, this technique is frequently accompanied by bile reflux and post-gastrectomy syndromes. Braun jejunojejunostomy is performed as an anti-reflux modification, but its benefits relative to B-II alone remain unknown. This systematic review and meta-analysis aims to assess the effects of B-II plus Braun anastomosis in comparison to B-II alone. We conducted a detailed search across PubMed, Scopus, and Web of Science from their inception up to November 2025 to identify pertinent studies comparing B-II+Braun anastomosis with B-II alone after distal gastrectomy. The potential risk of bias of the included studies was assessed using the ROBINS-I (Risk of Bias in Non-randomized Studies of Interventions) quality assessment tool. The meta-analysis was conducted using RevMan v.5.4 (Cochrane Collaboration, London, UK) employing a random-effects model. We identified eight retrospective studies (n = 2038 patients). Results of meta-analysis showed no difference in bile reflux incidence between B-II+Braun and B-II alone (RR: 0.95, 95% CI: 0.76-1.18). Early postoperative bile reflux showed a borderline reduction (RR: 0.57, 95% CI: 0.32-1.01). Anastomotic bleeding (RR: 0.94, 95% CI: 0.29-3.02) and fistula risk (RR: 1.17, 95% CI: 0.54-2.55) were comparable, and B-II+Braun had a longer operative time compared to B-II alone (mean difference: 19.07 minutes, 95% CI: 4.76-33.38). B-II with Braun enteroenterostomy offers no persistent benefits over the traditional B-II for bile reflux, anastomotic complications, or perioperative outcomes, and it increases operative time. This meta-analysis supports a personalized approach to reconstruction over the default Braun modification and underscores the need for further substantive comparative studies.

## Introduction and background

Due to its high incidence and mortality rate, gastric cancer is still one of the most significant health issues globally [1]. There were around one million cases diagnosed, in addition to the approximately 770,000 deaths attributed to this disease in 2020 alone [2]. For non-cardiac disease, distal (subtotal) gastrectomy with D1+/D2 lymphadenectomy is a standard curative surgical procedure, as per the Japanese Gastric Cancer Association (JGCA), European Society of Medical Oncology (ESMO), and recent phase 3 clinical trials [3-5]. Aside from its oncologic role, distal gastrectomy is still done for some other benign and functional diseases, including complicated or refractory peptic ulcer disease, gastric outlet obstruction, and surgical sequelae of prior upper gastrointestinal surgery, albeit with a more modern therapeutic approach [6]. Both for malignant and benign conditions, advances in perioperative medicine and improvements in long-term survival have refocused attention from purely oncologic outcomes to long-term functional outcomes, post-gastrectomy symptoms (PGS), and health-related quality of life (QoL), which have become necessary measures of success for these procedures [7].

Several reconstruction options are available after distal gastrectomy, such as Billroth I (B-I) and Billroth II (B-II), Roux-en-Y (RY), and uncut Roux-en-Y (uncut RY). Comparative studies and meta-analyses generally indicate that RY are less likely to cause bile reflux, remnant gastritis, and reflux esophagitis than B-I and B-II, and do not seem to lead to more complications, albeit they require more anastomoses and are also likely to cause Roux stasis [8]. On the other hand, B-II retains considerable popularity and utility, especially in uncomplicated cases when B-I is not feasible because a safe, tension-free gastroduodenostomy cannot be achieved (e.g., a shortened and/or fibrotic duodenal stump after chronic inflammation or ulcer-related scarring, or dense adhesions from prior upper gastrointestinal surgery), which may increase the risk of anastomotic complications [9]. However, classic B-II results in severe enterogastric biliary reflux, alkaline reflux gastritis (inflammatory injury of the remnant gastric mucosa caused by reflux of bile and pancreatic secretions), and afferent loop syndrome (functional or mechanical obstruction/kinking of the afferent limb leading to distension, pain, and bilious vomiting), which results in severe epigastric pain, bile vomiting, reflux bile, and mucosal damage to the remnant stomach [9]. Bile reflux has been proven to be an independent risk factor for gastric carcinoma, specifically post B-II [10]. These compromised outcomes have justified considering numerous technical variations of B-II to minimize or divert bile and to enhance functionality and long-term oncological outcomes.

Braun anastomosis is a side-to-side jejunojejunostomy performed between the afferent and efferent loops and is typically done 10 to 20 cm distal to the gastrojejunostomy. Braun's initial description in the 19th century explained this procedure as a method to divert bile and pancreatic secretions from the remnant stomach and alleviate afferent loop stasis [11]. Physiological and radionuclide studies have shown that a Braun enteroenterostomy reduces enterogastric bile reflux and improves symptoms associated with alkaline reflux after gastric surgery, despite a trade-off of lower bile reflux rates and an increased incidence of severe gastroparesis [12,13]. Studies have supported the biological plausibility of Braun's anastomosis as an anti-reflux modification of B-II, in which similar jejunojejunostomies have been employed in upper gastrointestinal reconstructions, such as in pancreaticoduodenectomy and biliary-enteric bypass [14].

Comparative research investigating Billroth II plus Braun jejunojejunostomy (B-II+Braun) versus Billroth II alone after gastrectomy continues to produce different outcomes. Some studies report that adding Braun may reduce early bile reflux, alkaline gastritis, and dumping, and improve nutritional outcomes and overall QoL [15]. On the other hand, other studies reported only temporary benefits and minimal advantages, raising the question of whether Braun should be included in routine procedures [16]. Considerable differences in surgical methods, patient populations, definitions of outcomes, and length of follow-up limit the reliability of individual studies, and the question has not been the focus of any systematic review or meta-analysis. Therefore, the current study aims to compare B-II+Braun and B-II with respect to bile reflux and postoperative outcomes.

## Review

Methods

Protocol and Registration

This study includes a systematic review and meta-analysis of outcomes, conducted following the instructions of the Cochrane Handbook for Systematic Reviews of Interventions (version 5.1.0) [17] and adhering to the established Preferred Reporting Items for Systematic Reviews and Meta-Analyses (PRISMA) guidelines for systematic reviews and meta-analyses [18]. An a priori protocol was developed and prospectively registered with the International Prospective Register of Systematic Reviews (PROSPERO: CRD420251246350).

Eligibility Criteria

Inclusion criteria: To include relevant studies pertinent to our research question, we chose studies published in English that satisfied the following PICOS (population, intervention, comparison, outcomes, study design) criteria: (i) population: adult patients undergoing distal gastrectomy for non-metastatic gastric cancer, complicated peptic ulcer or other indications; (ii) intervention: Billroth-II reconstruction with Braun jejunojejunostomy anastomosis (B-II+Braun); (iii) comparator: Billroth-II reconstruction without Braun anastomosis (B-II); (iv) outcomes: primary outcomes of interest included incidence of bile reflux, anastomotic bleeding, and anastomotic fistula. Secondary outcomes were operative time, intraoperative blood loss, postoperative complications (e.g., abdominal bleeding, gastric emptying dysfunction, and wound-related complications), and length of hospital stay. (v) Study design: Prospective or retrospective comparative studies.

Exclusion criteria: We excluded (i) non-comparative studies (including case reports, case series, or single-arm studies), (ii) letters to the editor, commentaries, and conference abstracts, (iii) studies including surgeries other than distal gastrectomy, (iv) studies comparing other reconstruction methods other than Billroth-II surgery, and (v) studies not published in English or without attached English translation.

Information Sources and Search Strategy

We conducted a literature search across three major databases (PubMed, Scopus, and Web of Science) from their inception through November 2025 to identify relevant records that met our eligibility criteria using the following search strategy: ("Billroth II" OR "Billroth-II" OR Billroth) AND ("Braun anastomosis" OR "Braun enteroenterostomy" OR "Braun") AND ("distal gastrectomy" OR "gastrectomy" OR "gastric surgery" OR "bariatric surgery"). No geographic restrictions were applied. Slight modifications were made to suit each database, and no other filters or restrictions were applied.

Study Selection and Data Extraction

Selection process: All records imported from the electronic databases searches were placed into EndNote (Clarivate, Philadelphia, PA) [19] for deduplication. The EndNote deduplicated library was then uploaded into the Rayyan platform [20] for screening and data management. The selection of the studies was conducted in two consecutive phases. (i) The first was a screening of titles and abstracts. (ii) The second was a full-text screening based on the previously defined inclusion and exclusion criteria. During each phase, two reviewers independently assessed records for eligibility in Rayyan, without visibility into each other's decisions. Disagreements were settled through discussion; when agreement could not be reached, the judgment of a third reviewer was considered decisive.

Data extraction: Data were extracted into a uniform pre-piloted Google sheet (Google, Mountain View, CA). At the study level, we documented the study ID (the first author's surname and year of publication), study design, country/region, population characteristics, intervention and comparator details (type of reconstruction and technical details), and sample size. At the individual patient level, we reported aggregated data on patient age and sex distribution and, when available, gastric cancer stage. For each study, we also extracted all results of primary and secondary predefined outcomes. Data extraction was done independently by two authors, and each extracted item was verified and cross-checked by two other authors to ensure completeness and precision. All discrepancies, if any, were discussed until a consensus was reached.

Assessment of Risk of Bias

The potential risk of bias of the included studies was assessed using the ROBINS-I (Risk of Bias in Non-randomized Studies of Interventions) tool [21]. This framework assesses the possible risk of bias across seven main domains: confounding, selection of participants, classification of interventions, deviations from intended interventions, missing data, measurement of outcomes, and selection of the reported result. Two independent reviewers conducted risk-of-bias assessments for each domain and determined an overall risk-of-bias rating for each study, classified as low, moderate, serious, or critical.

Statistical Analysis and Heterogeneity

Statistical analysis: Meta-analysis was conducted for outcomes reported in at least three studies using RevMan software v.5.4 (Cochrane Collaboration, London, UK) [22]. Dichotomous outcomes (e.g., anastomotic bleeding, fistula, bile reflux, abdominal bleeding, gastric emptying dysfunction, and wound complications) were pooled as risk ratios (RRs) with corresponding 95% confidence intervals (CIs) using the Mantel-Haenszel method. Continuous outcomes (operative time, intraoperative blood loss, and length of postoperative hospital stay) were synthesized as mean differences (MDs) with 95% CIs using the inverse-variance method. A random‐effects model was applied for all outcomes, as clinical and methodological heterogeneity across studies was anticipated.

Heterogeneity: Statistical heterogeneity was assessed using Cochran’s Q test (χ²) and quantified with the I² statistic, with values >50% considered to indicate substantial heterogeneity. All tests were two-sided, and a P-value <0.05 was considered statistically significant. Meta-regression and publication-bias assessments were prespecified; however, the small number of included studies (n = 8) precluded their reliable application, as these methods are typically underpowered when fewer than 10 studies are available.

Results

Study Selection

**Figure 1:**
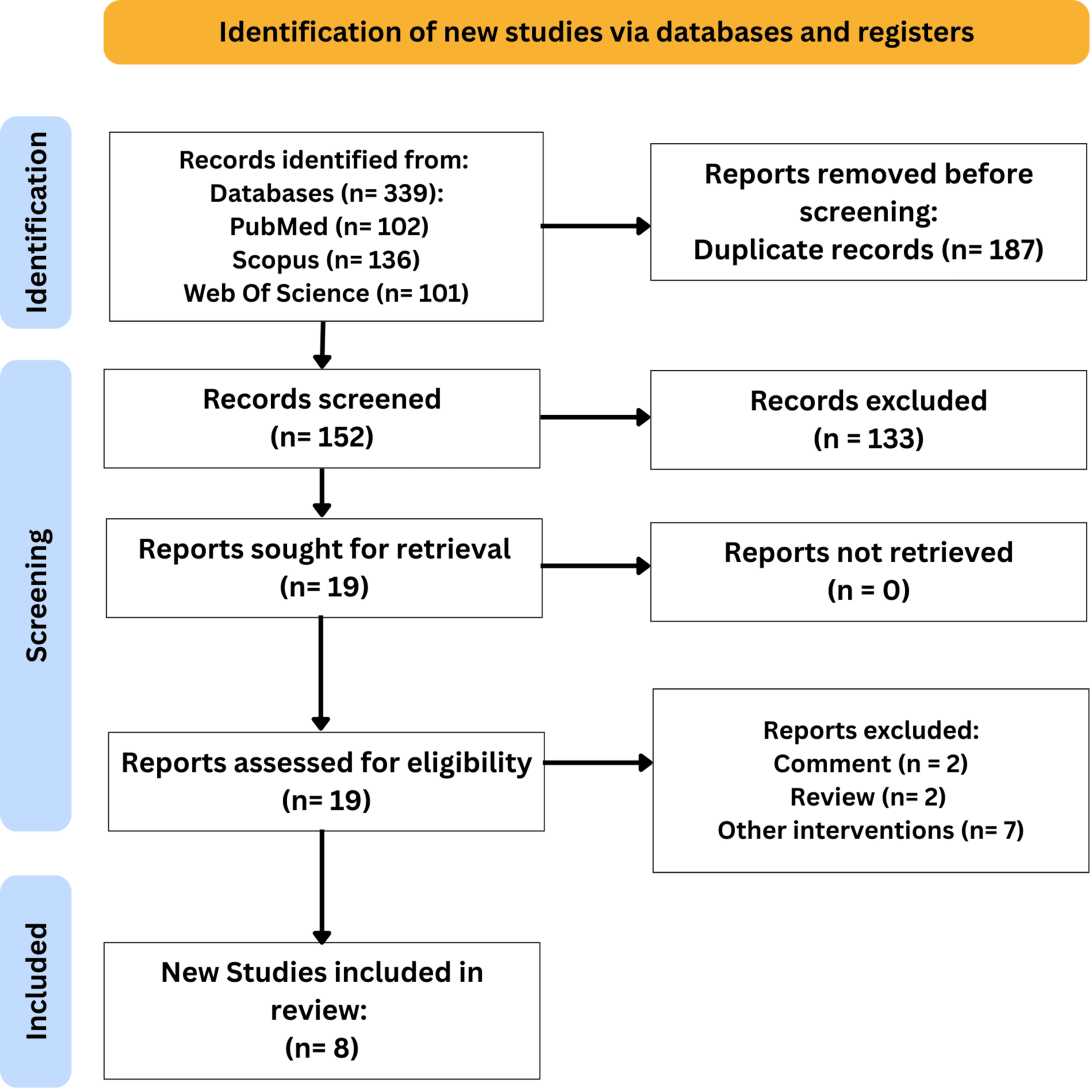
PRISMA flow diagram of the selection process. PRISMA: Preferred Reporting Items for Systematic Reviews and Meta-Analyses.

A total of 339 records were identified through database searches (PubMed = 102, Scopus = 136, Web of Science = 101). After removing 187 duplicates, 152 records were screened based on titles and abstracts, resulting in 133 exclusions. Full-text assessments were performed for 19 studies, leading to the exclusion of 11 studies (including two comments, two reviews, and seven with different interventions). In total, eight records [15,16,23-28] met the eligibility criteria and were included in the systematic review and meta-analysis. Figure 1 illustrates the PRISMA flow diagram summarizing the study selection process.

Characteristics of Included Studies

Eight retrospective comparative studies encompassing 2038 adults were eligible, with 1029 patients undergoing Billroth II with Braun anastomosis and 1009 undergoing Billroth II alone. Most studies were conducted in East Asia (China, South Korea, Taiwan), with one from Greece, and all included distal gastrectomy performed primarily for non-metastatic gastric cancer, with two series also including complicated peptic ulcer disease. The surgical approach across studies was open, laparoscopic, or mixed. Age was generally between the late 50s and early 70s; the majority of cohorts were men, and tumor-stage distributions were roughly similar across reconstruction groups in studies that reported stage. Table 1 presents a detailed overview of study designs and baseline clinical parameters across all included studies.

**Table 1:** Summary of included studies. B-II = Billroth II reconstruction; B-II+B = Billroth II reconstruction with Braun enteroenterostomy (Braun anastomosis); LN = lymph node; SD = standard deviation; n = number (with n (%) indicating number and percentage).

Study ID	Study design	Location	Population	Intervention	Control	Sample size		Age, mean ± SD		Sex, male, n (%)		Tumor stage					
												Stage 1		Stage 2		Stage 3	
						B-II+B	B-II	B-II+B	B-II	B-II+B	B-II	B-II+B	B-II	B-II+B	B-II	B-II+B	B-II
Wang et al. (2025) [16]	Retrospective analytical study	China	Adult patients aged 18–85 with successful completion of laparoscopic-assisted radical resection of distal gastric cancer	Laparoscopic lymph node dissection followed by (Billroth II + Braun)	LN dissection, then Billroth II reconstruction surgery	261	242	61.2 ± 8.2	60.8±10.9	173 (52.5)	161 (66.5)	51 (19.5)	56 (23.1)	108 (41.4)	91 (37.6)	102 (39)	95 (39.25)
Yu et al. (2023) [26]	Retrospective analytical study	China	Adult patients with no evidence of tumors invading adjacent organs or distant metastasis, who underwent open or laparoscopic radical distal gastrectomy	Open or laparoscopic Billroth II + Braun anastomosis	Billroth II without Braun	193	193	58.13 ± 12.18	58.24 ± 12.42	134 (69.4)	138 (71.5)	54 (28.0)	44 (22.8)	55 (28.5)	44 (22.8)	84 (43.5)	105 (54.4)
Chan et al. (2007) [28]	Retrospective analytical study	Taiwan	Patients who underwent distal gastrectomy for complicated peptic ulcers or stomach cancer	Billroth II + Braun anastomosis	Billroth II without Braun	12	29	68 ± 7.9	64.5 ± 13.5	8 (66.67)	17 (68)	6 (50%) patients had gastric cancer, and 6 (50%) patients had peptic ulcer in the Billroth ll + B group. 10 (34.5%) patients had gastric cancer, and 19 (65.5%) had a peptic ulcer in Billroth II					
Li et al. (2022) [25]	Retrospective analytical study	China	Adult patients aged 18–75 undergoing laparoscopic-assisted radical resection of distal gastric cancer	Billroth II + Braun anastomosis	Billroth II without Braun	84	59	57.2 ± 11.4	56.8 ± 9.9	56 (66.66)	41 (69.5)	7 (8.33)	5 (8.5)	30 (35.7)	16 (27.1)	23 (27.4)	12 (20.3)
Christodoulidis et al. (2024) [15]	Retrospective cohort study	Greece	Patients aged between 42 and 92 years old undergoing distal gastrectomy for gastric cancer or peptic ulcers	Billroth II + Braun anastomosis	Billroth II without Braun	28	74	70.41	70.75	21 (75)	44 (59.4)	5 patients were diagnosed with gastrointestinal stromal tumor, 6 patients with gastric ulcer, and all the remaining patients suffered from gastric adenocarcinoma					
Fu et al. (2024) [23]	Retrospective cohort study	China	Patients undergoing a totally laparoscopic distal gastrectomy	Laparoscopic lymph node dissection followed by Billroth II + Braun )	LN dissection, then Billroth II	93	68	59.27 ± 11.43	61.05 ± 9.66	63 (67.74)	44 (67.69)	46 (49.5)	36 (52.9)	19 (20.4)	10 (14.7)	4 (4.3)	2 (2.9)
Wang et al. (2024) [24]	Retrospective real-world study	China	Patients with adenocarcinoma confirmed by preoperative gastroscopy and histopathological biopsy, necessitating radical distal gastrectomy without distant metastasis	Open or laparoscopic Billroth II + Braun anastomosis	Billroth II without Braun	192	185	63.5 ± 7.0	61.8 ± 12.8	115 (59.9)	107 (57.8)	80 (41.7)	76 (41.1)	22 (11.5)	17 (9.2)	35 (18.2)	37 (20.0)
Kim et al. (2025) [27]	Retrospective cohort study	South Korea	Patients aged ≥18 years with localized gastric cancer who underwent distal gastrectomy	Billroth II + Braun anastomosis	Billroth II without Braun	166	159	60.7 ± 11.4	60.8 ± 10.2	110 (66.3)	110 (69.2)	105 (63.3)	105 (66.0)	35 (21.1)	29 (18.2)	26 (15.7)	25 (15.7)

Risk of Bias and Quality Assessment

**Figure 2:**
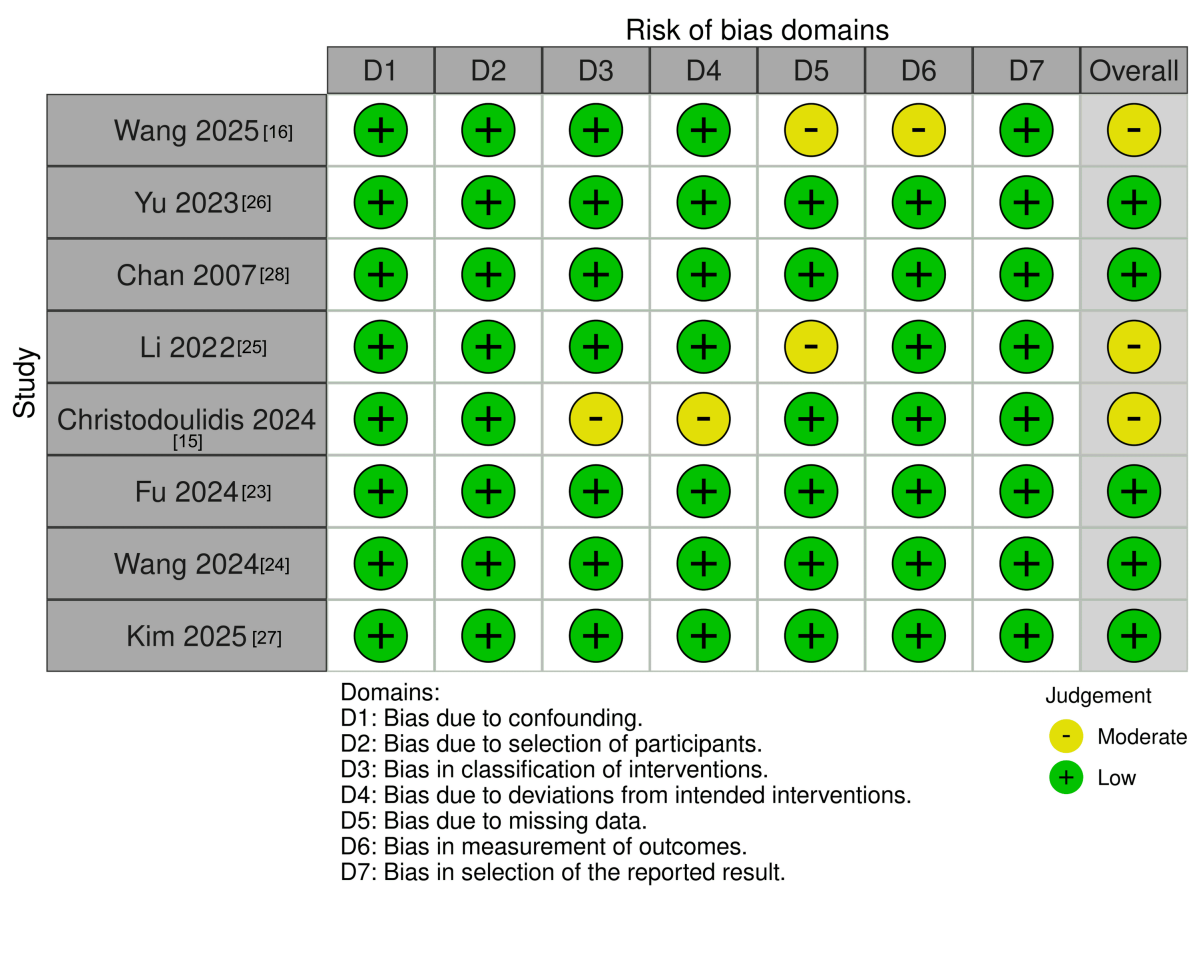
Risk of bias assessment graph using ROBINS-I tool. ROBINS-I = Risk of Bias in Non-randomized Studies of Interventions.

Overall, the risk-of-bias assessment of the included studies using the ROBINS-I tool indicated good-quality evidence. Three studies [15,16,25] were judged to have a “Moderate” risk of bias, whereas the remaining five studies [23,24,26-28] were judged to have a “Low” risk of bias. No study was judged to be at critical or serious risk of bias. Figure 2 visually depicts the domain-specific and overall risk assessments for all studies included.

Primary Outcomes

**Figure 3:**
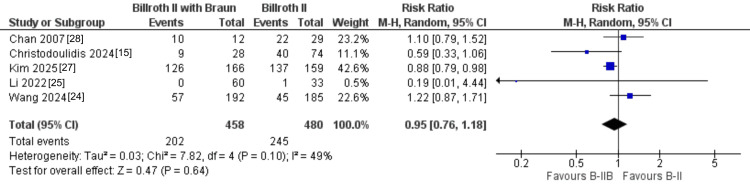
Forest plot of the pooled risk ratio of bile reflux comparing Billroth II with Braun anastomosis (B-II+Braun) versus standard Billroth II (B-II).

**Figure 4:**
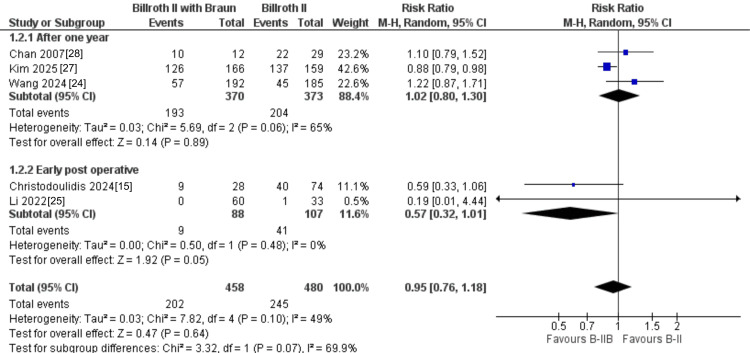
Subgroup analysis of bile reflux according to timing of assessment (early postoperative vs. ≥1 year).

Bile reflux: Bile reflux was evaluated in five studies, including 938 patients. Although the absolute rate of bile reflux tended to be lower after B-II+Braun than after B-II alone (202/458 vs. 245/480 events), the difference was not statistically significant (RR = 0.95, 95% CI: 0.76-1.18; P = 0.64), with moderate heterogeneity across studies (I² = 49%) (Figure 3). In the subgroup analysis according to timing of assessment, B-II+Braun showed a trend toward reduced early postoperative bile reflux (RR = 0.57, 95% CI: 0.32-1.01; P = 0.05), whereas no difference was observed at ≥1 year of follow-up (RR = 1.02, 95% CI: 0.80-1.30; P = 0.89). However, the test for subgroup differences did not reach statistical significance (P = 0.07), so this potential time-dependent effect should be interpreted with caution (Figure 4).

**Figure 5:**
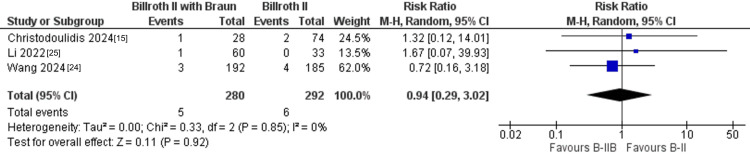
Forest plot of anastomotic bleeding comparing B-II+Braun versus B-II. B-II = Billroth II.

**Figure 6:**
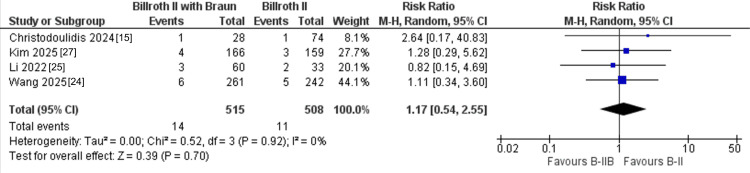
Forest plot of anastomotic fistula comparing B-II+Braun versus B-II. B-II = Billroth II.

Anastomotic complications: Regarding anastomotic complications, three studies including 572 patients compared the incidence of anastomotic bleeding between B-II+Braun and standard B-II reconstruction. The pooled analysis showed no significant difference between the two techniques (5/280 vs. 6/292 events; RR = 0.94, 95% CI: 0.29-3.02; P = 0.92), with no evidence of heterogeneity (I² = 0%) (Figure 5). Similarly, four studies with 1,023 patients reported anastomotic fistula, and the meta-analysis again demonstrated no significant difference between B-II+Braun and B-II (14/515 vs. 11/508 events; RR = 1.17, 95% CI: 0.54-2.55; P = 0.70; I² = 0%) (Figure 6).

Secondary Outcomes

Hemorrhagic outcomes (abdominal bleeding): Three studies, including 872 patients, reported abdominal bleeding, and the pooled analysis showed no significant difference between B-II+Braun and B-Ⅱ alone (7/452 vs. 5/420 events; RR = 1.33, 95% CI: 0.43-4.17; P = 0.62; I² = 0%) (Appendix, Figure S1).

Operative metrics (intraoperative blood loss and operative time): Intraoperative blood loss was evaluated in seven studies (993 vs. 954 patients), and there was no significant difference between the two groups (MD = −2.36 mL, 95% CI: −11.52 to 6.80; P = 0.61), with moderate heterogeneity (I² = 54%) (Appendix, Figure S2). Operative time was reported in seven studies (993 vs. 954 patients) and was significantly longer in the B-II+Braun group compared with B-Ⅱ (MD = 19.07 minutes, 95% CI: 4.76-33.38; P = 0.009; I² = 92%) (Appendix, Figure S5).

Postoperative recovery (gastric emptying dysfunction and length of stay): Three studies with 1,050 patients reported postoperative gastric emptying dysfunction; the incidence tended to be lower following B-II+Braun than B-Ⅱ alone (5/547 vs. 12/503 events), but this difference was not statistically significant (RR = 0.42, 95% CI: 0.15-1.16; P = 0.09; I² = 0%) (Appendix, Figure S3). Length of postoperative hospital stay was reported in six studies (933 vs. 921 patients). Patients undergoing B-II+Braun reconstruction tended to have a shorter stay, but the difference was not statistically significant (MD = −1.30 days, 95% CI: −2.85 to 0.25; P = 0.10), with substantial heterogeneity (I² = 93%) (Appendix, Figure S4).

Wound-related complications: Three studies, including 1,205 patients, assessed wound-related complications, and no significant difference was found between B-II+Braun and B-Ⅱ (9/619 vs. 14/586 events; RR = 0.64, 95% CI: 0.28-1.50; P = 0.31; I² = 0%) (Appendix, Figure S6).

Discussion

This systematic review and meta-analysis evaluated the comparative outcomes of the Billroth II reconstruction with Braun enteroenterostomy versus the Billroth II alone techniques after distal gastrectomy. In a pooled analysis of nearly 2,000 patients, the addition of Braun enteroenterostomy did not significantly reduce bile reflux incidence overall compared with Billroth II alone. Subgroup analysis suggested a potential trend toward decreased reflux during the early postoperative period. However, this trend did not reach statistical significance and was not sustained beyond the first postoperative year. Rates of anastomotic fistula and bleeding were low and comparable between the two techniques. Regarding secondary outcomes, there were no noticeable differences in the incidence of postoperative hospital stay, wound complications, intraoperative bleeding, gastric emptying dysfunction, or abdominal bleeding. On the contrary, the operative time was much longer in patients with a Braun enteroenterostomy. Taken together, this meta-analysis showed that the addition of a Braun anastomosis increased operative complexity without apparent clinical benefit.

The main reason to include a Braun enteroenterostomy is to redirect bile from the gastric remnant and to alleviate duodenogastric reflux. Braun diversion has been documented through classical physiological and radionuclide studies to reduce the reflux gastritis and improve symptoms associated with bile reflux following gastrectomy or gastrojejunostomy. Braun diversion has been considered a simpler alternative to the Roux-en-Y diversion in this medical context [29]. Recent studies have yielded mixed, non-robust results; a single-center series reported that B-II+Braun has a notably lower rate of postoperative reflux gastritis, supporting its hypothesized, biologically plausible effect [15]. Another extensive center study showed a slight early reduction in bile reflux rates, yet this effect was short-lived [27]. Our meta-analysis clarifies that although early anti-reflux signs of effect from the Braun reconstruction may be present, they are modest, do not reach statistical significance, and are, therefore, unlikely to translate into a meaningful clinical benefit. Therefore, the Braun enteroenterostomy technique cannot be recommended as a universally superior method for reflux control in all patients undergoing distal gastrectomy.

Another key finding from this meta-analysis is that the incorporation of a Braun enteroenterostomy did not lead to a statistically significant increase in anastomotic complications. There was no statistically significant difference among those complications, which include anastomotic bleeding and fistulas, in both groups. Although the event rates were low and the confidence intervals were very wide, which would point to a lack of power sufficient to detect a statistically significant difference, the consistency of the null results from various studies suggests that the additional jejunojejunostomy does not significantly impact the closure of the anastomosis in the gastrojejunostomy or the anastomosis of the duodenal stump. This is also supported by other studies that have found similar leak and bleeding rates when comparing Billroth II with Braun to more advanced, complex anti-reflux procedures like Roux-en-Y, in both single-center reports and more recent comparative meta-analyses [30].

The secondary outcomes further strengthen the evidence that the perioperative safety of B-II and B-II+Braun is approximately the same. The rates of abdominal bleeding, intraoperative blood loss, and complications of the wounds were the same, affirming the idea that the addition of Braun does not significantly raise the risk of early surgical trauma or postoperative wound morbidity. Meta-analysis results showed a trend toward reduced gastric emptying and shorter hospital stays in the B-II+Braun arm, despite the lack of strong evidence from the overall data, which aligns with the idea that changes in jejunal flow and bile diversion can improve gastric emptying and accelerate recovery in some patients [31]. However, these signals are imprecise, narrow in scope, unreliable, and highly sensitive to center-level practice patterns regarding surgery, postoperative diet, discharge criteria, and minimally invasive techniques [32]. They lack significant, clinically meaningful differences and should instead be considered hypothesis-generating rather than changing practice in the future. Conversely, the influence on operative time is more evident both statistically and clinically. In the studies, B-II+Braun had a significant increase in operative time compared with B-II without Braun. This is in line with the general reconstructive literature, where any further anastomosis, whether it be Braun enteroenterostomy, Roux-en-Y limb construction, or uncut Roux-en-Y, lengthens the operative time [33].

This study further builds upon the existing literature on reconstruction after distal gastrectomy and its associated alterations. Several network meta-analyses and meta-analyses have assessed and compared the Billroth I, Billroth II, and the Roux-en-Y reconstructions and have noted Roux-en-Y to be the most effective surgical technique in reducing bile reflux and remnant gastritis, even though it does require higher levels of surgical skill and comes with complications such as internal hernia or Roux stasis [33,34]. Billroth I, on the other hand, offers shorter operative and recovery times but comes with a higher incidence of reflux [35]. Other studies comparing Billroth II with Roux-en-Y also demonstrate that Roux-en-Y is superior with respect to remaining gastritis, reflux esophagitis, reflux symptoms, and does not incur any significant safety costs, which solidifies its position as the most "aggressively anti-reflux" option [36]. Considering the intra-cohort and inter-cohort comparisons of Billroth II with and without Braun and the formal aggregating of early and late bile reflux data, our review can demonstrate that the Braun modification does not imply any extra added anti-reflux effect to B-II or closes the gap between B-II and Roux-en-Y.

Clinical Implications

The most recent evidence-based recommendations for the management of gastric cancer in the Japanese Gastric Cancer Treatment Guidelines 2018 (5th edition) and its 2021 update, which include Billroth I, Billroth II, and Roux-en-Y, consider these options for reconstruction after distal gastrectomy, while leaving the choice to the surgeon's discretion based on preferences, location of the tumor, and specialty of the institution, without suggesting any of them as a gold standard formation [3,37]. This meta-analysis refines the idea as follows: Billroth II with Braun consistently prolongs operative time and does not provide a clear, long-term benefit for bile reflux or perioperative outcomes. Any early anti-reflux signal observed is low and clinically insignificant, given the lack of statistical significance. Therefore, adding a Braun anastomosis will not make a demonstrable difference compared with standard B-II.

Strengths and Limitations

A few different strengths stand out in this review. To our knowledge, this is the first meta-analysis to quantify the additional effect of including a Braun enteroenterostomy in Billroth II reconstructions, rather than simply pooling it with other reconstructions. This incorporates data from more than 2000 patients and is better able to refine estimates of the complications of bile reflux and anastomosis, as well as other critical perioperative outcomes. Most notably, the time-based analysis of bile reflux and the accompanying analyses of operative time and recovery endpoints provide a reasonable estimate of the short- and long-term trade-offs in the Braun modification.

The accompanying evidence of this meta-analysis has several limitations due to all compositions being retrospective, non-randomized cohorts (eight studies predominantly made of East Asian subjects, lacking geographically diversity), leading to results being open to confounding by indication, selection bias, and center/surgeon effects beyond correction by pooling. The follow-up may also have been limited in a way that insufficiently characterized reflux-related morbidity and quality of life outcomes reported by patients. Moreover, there was also significant heterogeneity in some of the secondary outcomes (i.e., length of stay and operative time), suggesting variability in surgical approach (open vs. laparoscopic). Variability in intraoperative and postoperative measures and discharge policies also affects the degree of pooling estimates and makes them less generalizable. Publication bias assessment and meta-regression were prespecified; however, the small number of included studies (n = 8) precluded reliable application of these methods, as they are typically underpowered when fewer than 10 studies are available.

## Conclusions

Based on this systematic review and meta-analysis, the Braun anastomosis added to standard Billroth-II reconstruction did not reduce bile reflux compared with standard Billroth II alone. Early postoperative signals showed that B-II+Braun may have a modest effect; however, this effect was statistically and clinically non-trivial. B-II+Braun showed comparable results regarding postoperative complications and length of hospital stay. However, B-II+Braun resulted in a longer operative time than B-II alone.

## Figures and Tables

**Figure 7:**
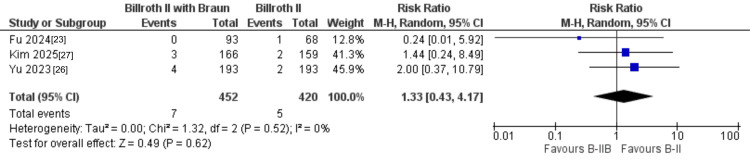
Forest plot of abdominal bleeding comparing Billroth II with Braun anastomosis (B-II+Braun) versus standard Billroth II (B-II) after distal gastrectomy.

**Figure 8:**
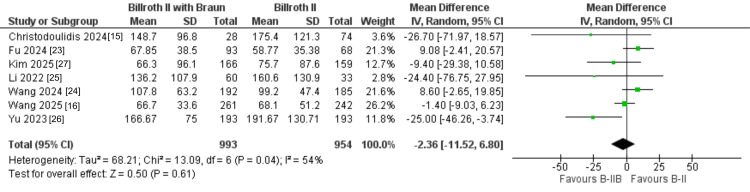
Forest plot of intraoperative blood loss (mL) comparing B-II+Braun versus B-II. B-II = Billroth II.

**Figure 9:**
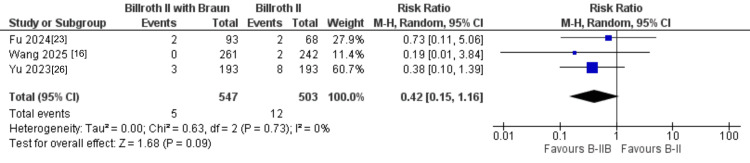
Forest plot of postoperative gastric emptying dysfunction comparing B-II+Braun versus B-II. B-II = Billroth II.

**Figure 10:**
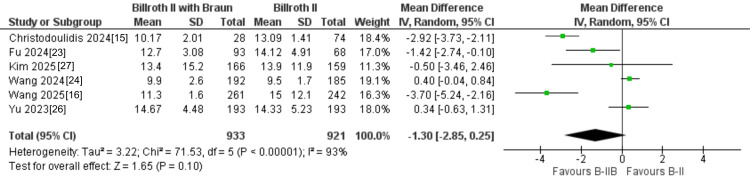
Forest plot of length of postoperative hospital stay (days) comparing B-II+Braun versus B-II. B-II = Billroth II.

**Figure 11:**
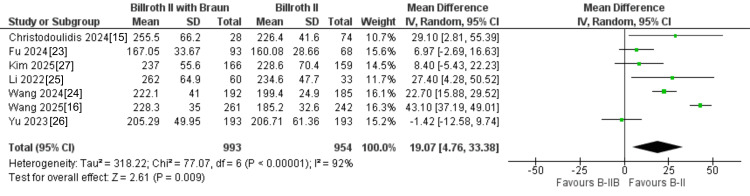
Forest plot of operative time (minutes) comparing B-II+Braun versus B-II. B-II = Billroth II.

**Figure 12:**
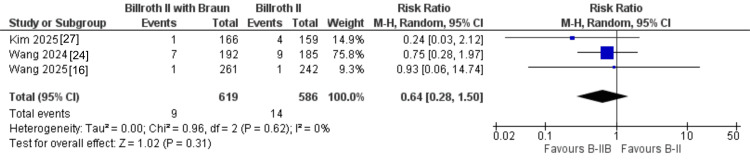
Forest plot of wound-related complications comparing B-II+Braun versus B-II after distal gastrectomy. B-II = Billroth II.

## Appendices

Figure S1

 Figure S2

Figure S3

Figure S4

Figure S5

Figure S6
